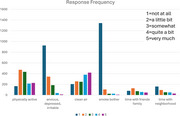# Analysis of ecological momentary assessment data from the first completed cohort

**DOI:** 10.1002/alz70858_101243

**Published:** 2025-12-25

**Authors:** Diane J Cook

**Affiliations:** ^1^ Washington State University, Pullman, WA, USA

## Abstract

**Background:**

Understanding the relationship between behavior, environment, and dementia risk is critical for assessing well‐being and designing interventions. Smartwatch sensor data can provide objective insights into these relationships. This study examines digital markers associated with EMA responses in a cohort from rural Florida.

**Method:**

Data were collected for 17 participants who wore Apple Watches daily. The watches used an in‐house app that queried participants 4x/day about physical activity, anxiety, air quality, and smoke exposure, rated on a scale from 1 (not at all) to 5 (very much). Each day, participants reported time spent with friends/family and neighbors. They also completed our custom 45‐second smartwatch‐based *n*‐back shape test to measure cognition. Home was defined as the most frequent location each morning, and social isolation was estimated as the percent time spent at home. We analyzed EMA responses, *n*‐back scores, and their correlations with social isolation.

**Result:**

Participants wore watches for Mean = 356.78 hours (*SD* = 189.45) and responded to Mean = 89.18 prompts (*SD* = 51.36). Response distributions were active: *M* = 2.91, *SD* = 1.22; anxious: *M* = 1.59, *SD* = 0.86; air: *M* = 3.36, *SD* = 1.39; smoke: *M* = 1.18, *SD* = 0.60; friends/family: *M* = 2.66, *SD* = 1.29; neighbors: *M* = 2.20, *SD* = 1.30. *N*‐back scores were *M* = 19.71 (*SD* = 4.84). Small correlations were observed between *n*‐back scores and activity level (*r* = ‐0.34), anxiety (*r* = 0.30), and time with friends/family (*r* = 0.18). Activity was correlated with neighbor time (*r* = 0.39). Additionally, friend/family time correlated positively with neighbor time (*r* = 0.53) and negatively with time spent at home (*r* = ‐0.11), while neighbor time correlated positively with time spent at home (*r* = 0.14). The negative correlation between *n*‐back score and time spent at home was very small (*r* = ‐0.04).

**Conclusion:**

Smartwatch data that include a real‐time measure of cognition offer valuable insights into the relationship between self‐reported states, behavior, and air quality. Larger, more diverse samples are needed to enhance the generalizability of these findings.